# Gastric Band Port Site Fixation: Which Method Is Best?

**DOI:** 10.1155/2015/701689

**Published:** 2015-01-28

**Authors:** Corinne E. Owers, Sarah M. Barkley, Roger Ackroyd

**Affiliations:** Department of Upper GI and Bariatric Surgery, Sheffield Teaching Hospitals NHS Foundation Trust, Herries Road, Sheffield S5 7AU, UK

## Abstract

Laparoscopic adjustable gastric banding is a popular and successful bariatric surgical technique. Although short-term complications are few in number, long-term complications are more common. One such complication is flippage of the gastric band port. This study compares three popular methods of port fixation and demonstrates that fixation with nonabsorbable mesh helps to prevent port flippage when compared to other techniques, reducing the need for repositioning operations.

## 1. Introduction

Laparoscopic gastric banding is a popular and effective bariatric surgical technique, shown to help obese patients lose excess weight, reduce comorbidities, and improve health related quality of life. A relatively simple procedure, numerous techniques have been described for placing a gastric band, with varying degrees of success in terms of long-term results. Along with the choice of technique used for the intra-abdominal aspect of the operation, the technique for port placement and fixation also has a bearing on long-term results. Different operative techniques are used to fix ports in place; we aimed to assess which method of port fixation yielded superior results in terms of reduced complications and reduced reoperation rates.

## 2. Methods

A retrospective case note review was performed for all the gastric banding procedures performed by one consultant surgeon (RA) since 2001. Over that time period, both the Allergan LAP-BAND and the Ethicon Swedish adjustable gastric band have been used in 4 different hospitals by the same surgeon.

Intra-abdominal placement of the gastric bands was performed using a pars flaccida approach in each case. Patients were placed in a steep reverse Trendelenburg position, pneumoperitoneum induced and the liver retracted using a Nathanson retractor. The pars flaccida was opened, the gastric band passed through a retrogastric tunnel and fastened anteriorly. A cuff of stomach was wrapped over the gastric band and sutured in an attempt to prevent migration or slippage of the band. The gastric band tubing was pulled through one of the 10 mm port sites and the instruments withdrawn.

A number of port fixation techniques were used.

### 2.1. Fixation of Ethicon Bands

All Ethicon Swedish laparoscopic adjustable gastric band ports were fixed using a Velocity port fixation device. A skin incision was made below the left costal margin and a skin pocket was created. The Velocity device containing the gastric band port was placed firmly against the anterior abdominal wall and the device fired fixing it firmly to the rectus sheath.

### 2.2. Fixation of Allergan Bands

Until September 2009, all Allergan LAP-BAND gastric band ports were fixed using direct suture fixation. Once creating a subcutaneous pocket below the left costal margin, four nonabsorbable sutures were placed in four quadrants around the port. The port was then sutured to the anterior abdominal wall and the tubing was connected.

**Figure 1:**
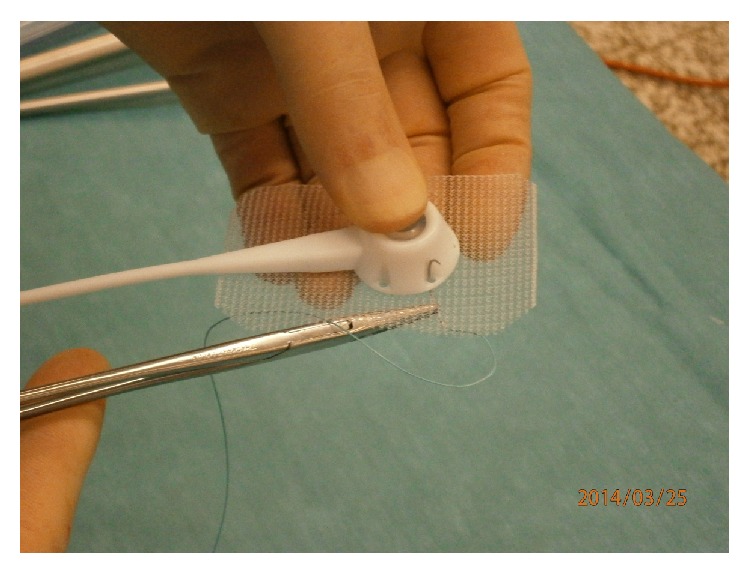
Attachment of port to mesh.

**Figure 2:**
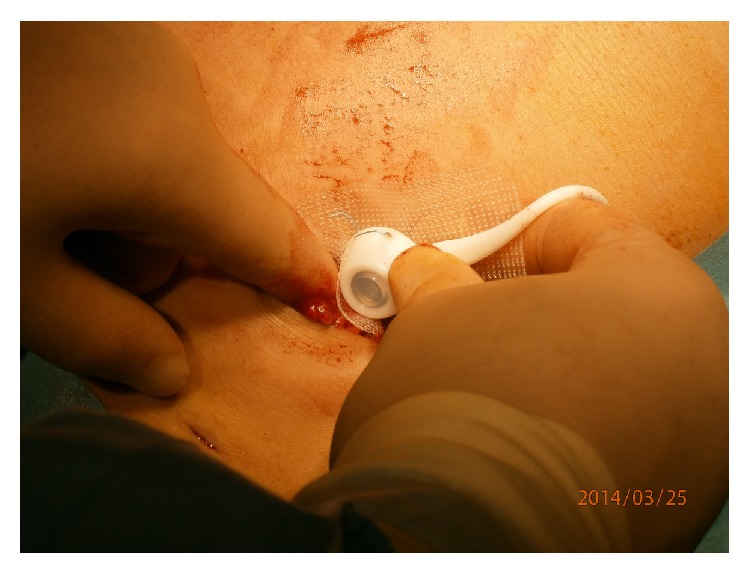
Placement of port and mesh into subcutaneous pocket.

Since September 2009, all Allergan LAP-BAND ports were sutured to a piece of nonabsorbable mesh and then placed into a subcutaneous pocket below the left costal margin without fixation of the mesh to the anterior rectus sheath (see Figures 1 and 2).

All bands were adjusted at 4–6 weeks under radiological guidance.

A Chi squared analysis was performed to see if there was a statistically significant difference between the different methods of port site fixation.

## 3. Results

Between January 2001 and January 2014, 1243 gastric bands were placed. See Table 1.

### 3.1. Ethicon Bands

Nine hundred and eighty-five Ethicon Swedish adjustable gastric bands were fitted using a Velocity fixation device. Of these, a total of 58 ports (5.8%) were flipped, making access for band adjustments impossible, even with the aid of fluoroscopy or ultrasound. Each of these ports therefore needed repositioning. In most cases, the skin overlying the band port was incised and tissue was dissected down to the anterior abdominal wall. The port was refixed to the fascia of the abdominal wall using a Velocity device, onto virgin tissue where possible. Skin was closed using staples and the band left for approximately 4–6 weeks before the first adjustment performed. In two patients, the band port was flipped a second time, requiring a second repositioning operation.

### 3.2. Allergan Bands

Before September 2009, 45 Allergan LAP-BAND were placed using a suture port fixation technique. Of these, two ports (4.4%) were flipped and required repositioning in order to be able to adjust the gastric band. In these cases, the ports were repositioned using the mesh technique, creating a subcutaneous pocket in virgin tissue into which the mesh with the port attached could be placed. Skin was then closed using skin clips, and again, band fills were performed after 4–6 weeks.

Since September 2009, 303 bands were fitted using the nonabsorbable mesh port fixation technique. In this group, only one patient (0.3%) suffered a port flippage which required repositioning.

Use of the mesh technique yielded a statistically significant reduction in port site flippages which require reoperation when compared to both the suture technique for the Allergan LAP-band and the Velocity technique for the Ethicon band (see Table 2). There was no statistical significance between the suture and Velocity devices when compared to each other.

## 4. Discussion

Gastric banding has been one of the most common bariatric surgical techniques of recent times. Though successful in terms of weight loss, laparoscopic adjustable gastric band surgery has been losing popularity over the last few years, partly due to the higher risk of long term complications when compared to the other bariatric procedures such as the sleeve gastrectomy and the roux-en-y gastric bypass [1]. Common complications experienced by patients with gastric bands are problems with the port, the port site, or port tubing. As well as the more serious complications of port site infection and erosion through the skin, both of which require removal of the gastric band port and potential replacement at a later date after healing has occurred, a less serious but annoying problem is that of port site flippage.

Ports can occasionally flip, either partially so that they lie on their side, or completely, so that the pierceable membrane lies against the abdominal wall. Although in some cases it may be possible to pinpoint the position of the port more easily and access it with the aid of ultrasound or fluoroscopy, in other cases port repositioning or replacement may be required.

Port site flippage has been reported in numerous studies with an incidence of 2–11% [1–4]. Once flipped, the port becomes difficult to access during band adjustments and sometimes impossible, even with radiological assistance [3, 5]. Numerous port fixation methods have been tried in an attempt to prevent port flippage. Some gastric band manufacturers have created a fixation device, where the port is fixed to the abdominal wall; these devices include the Ethicon Velocity and the Allergan RapidPort EZ Port Applier. Ports can also be fixed with a nonabsorbable suture such as prolene.

Port sites can become infected following gastric banding surgery [6]. Although this is often caused by band erosion, primary infection of the port site can occur due to surgical technique or aseptic band adjustments [7]. Once a mesh becomes infected, it is often very difficult to treat even with antibiotics, and removal is often necessary [8]. Given the need to access the gastric band port following LAGB placement, likelihood of mesh infection may seem high. However, in this study, no infections were seen in the mesh fixation group during the duration of this study, demonstrating that even 5 years following placement, mesh infection is not a significant problem as long as due care is taken.

The mesh fixation technique described for the purposes in this paper has been described by other authors, also demonstrating a low incidence of port flippage [9, 10].

This retrospective study has demonstrated the potential use of mesh port site fixation. Further prospective studies are now necessary to evaluate this technique.

## 5. Conclusion

Mesh fixation of the gastric band port can significantly reduce the incidence of port site flippage, minimising the need for port repositioning operations.

## Figures and Tables

**Table 1:** Numbers of ports fixed with each technique and numbers of flippages requiring repositioning.

Band type	Number	Flippage (requiring repositioning)	Percentage %
Swedish LAGB	985	58	5.8
Allergan pre 2009	45	2	4.4
Allergan post 2009	303	1	0.3

**Table 2:** Statistical significance comparing fixation techniques.

Technique	*P* value
Mesh versus suture	<0.001
Mesh versus velocity	<0.001
Suture versus velocity	0.68
